# Low Sex Hormone-Binding Globulin Levels Associate with Prediabetes in Chinese Men Independent of Total Testosterone

**DOI:** 10.1371/journal.pone.0162004

**Published:** 2016-09-01

**Authors:** Hui Zhu, Ningjian Wang, Bing Han, Qin Li, Yi Chen, Chunfang Zhu, Yingchao Chen, Fangzhen Xia, Zhen Cang, Meng Lu, Chi Chen, Dongping Lin, Yingli Lu

**Affiliations:** Institute and Department of Endocrinology and Metabolism, Shanghai Ninth People’s Hospital, Shanghai JiaoTong University School of Medicine, Shanghai 200011, China; Shanghai Diabetes Institute, CHINA

## Abstract

**Objective:**

The association ns between prediabetes and androgens have been rarely reported, especially in Chinese men. We aimed to investigate whether androgens were associated with the prevalence of prediabetes diagnosed with new American Diabetes Association criteria in Chinese men and then to assess which androgen value was the most relevant factor.

**Methods:**

A total of 2654 men (52.6±13.4 years old) were selected. Serum total testosterone (TT), sex hormone-binding globulin (SHBG) and free testosterone (FT) were measured. Covariance analysis of different androgen values were performed in age subgroups. Multinomial logistic regression was used for the association of TT, SHBG and FT with prediabetes and diabetes, as well as prediabetes in age subgroups.

**Results:**

According to ADA new criteria, normoglycemia, prediabetes, and diabetes were diagnosed in 1405, 907 and 342 men, respectively. In covariance analysis, SHBG of prediabetes were found lower than that of normoglycemia but higher than that of diabetes (*P* <0.05). In multinomial logistic regression, serum TT and SHBG were inversely associated with prediabetes and diabetes. While, after full adjustment for age, residence area, economic status, waist circumference, metabolic factors, other two androgen values and HOMA-IR, only the associations of SHBG with prevalence of prediabetes and diabetes persisted statistically significant, especially in the elderly with prediabetes (all *P* for trend <0.05).

**Conclusions:**

Serum androgen was inversely associated with prediabetes and diabetes in Chinese men. Low serum SHBG was the most relevant factor for prediabetes and diabetes. Whether it is an independent predictor for incident prediabetes in Chinese men needs further explorations.

## Introduction

Type 2 diabetes mellitus (T2DM), the major component of diabetes mellitus, is a highly prevalent complex chronic disease in China, where the age-standardized prevalence has rapidly increased from 9.7% in 2008 to 11.6% in 2010 [1]. However, unlike the USA, which has the similar high rate of T2DM as well as the high rate of obesity, China has much lower rate of overweight and obesity [2, 3]. On the other hand, the phenomena that both serum testosterone levels of young Chinese men and the rates of hormone related cancers in older Chinese men are significantly lower than those of Western populations [4], suggesting that low testosterone may be one of the potential risk factors contributing to the prevalence of T2DM in China other than overweight and obesity. Previous epidemiological studies have shown that diabetes is associated with testosterone deficiency in male populations. It has been reported that approximately 25% to 40% of diabetic men have low testosterone levels [5, 6], as well as male patients with hypogonadism, such as Klinefelter’s syndrome, have an increased incidence of diabetes [7]. Serum total testosterone (TT) is composed of free testosterone (FT) (2–3%), albumin-bound (20–40%), and sex hormone-binding globulin (SHBG) bound testosterone (60–80%). However, the associations between serum TT, FT and SHBG and T2DM in male populations were not identical in previous investigations [6, 8–12].

Prediabetes, the stage between normal glucose metabolism and diabetes, is characterized by β-cell dysfunction and increased insulin resistance [13]. The prevalence of prediabetes in China has remarkably increased to 50.1% in 2010 [1]. Impaired fasting glucose (IFG) and impaired glucose tolerance (IGT) are used in the diagnosis of prediabetes in most countries, while the glycated hemoglobin A1c (HbA1c) of 5.7% to 6.4% is considered as a new diagnostic criteria by the American Diabetes Association (ADA) recently [14]. Prediabetes identified by IFG, IGT, or the new HbA1c criteria may be caused by different mechanisms and represent different features of metabolic derangement [15, 16]. However, few studies have investigated the associations between androgens and prediabetes in men, especially in Chinese male populations [17].

The objective of the present study was to investigate whether serum TT, FT or SHBG levels was associated with the prevalence of prediabetes diagnosed with new ADA criteria in male populations from the SPECT-China study and then assess that which kind of androgens was the most related factor for prediabetes.

## Materials and Methods

### Study design and subjects—SPECT-China study

SPECT-China is a population-based cross-sectional survey on prevalence of metabolic diseases and risk factors in East China, which is made up of Shanghai and 7 provinces with a population of approximately 395 million in 2011, accounting for 29.2% of people in China. 99.5% of residents are Han Chinese. Registration number is ChiCTR-ECS-14005052, www.chictr.org. This study was performed in Shanghai, Zhejiang and Jiangxi Province from February to June 2014. Adults aged 18 years old and above who were Chinese citizens and lived in current residence for 6 months or longer were recruited for this study. Those who had acute illness, severe communication problems or refused to participate in the study were excluded. A total of 7200 people participated in this investigation. The participants who were younger than 18 years old (n = 6) or had missing lab results (n = 183) and questionnaire data (n = 112) were excluded from this study. Finally, 6899 subjects were enrolled, of whom 2654 participants were male subjects without hormone replacement therapy. Details on the sampling frames and the cohort examination procedures have been published elsewhere [18]. Thus, the current analyses were based on a total of 2654 men who met all the inclusion criteria. All participants provided written informed consent before data collection, and the study was approved by the Ethics Committee of Shanghai Ninth People’s Hospital, Shanghai JiaoTong University School of Medicine.

### Clinical, anthropometric, and laboratory measurements

In every study site, all the data collection was performed by the same staff group from Department of Endocrinology in Shanghai Ninth People’s Hospital affiliated to Shanghai Jiaotong University School of Medicine. They were trained according to a standard protocol that made them familiar with the specific tools and methods used. Trained staff used a questionnaire to collect information on demographic characteristics, medical history and lifestyle risk factors. Body weight, height, waist circumference and blood pressure were measured with the use of standard methods as described previously[1]. Body mass index (BMI) was calculated as weight in kilograms divided by height in meters squared. Insulin resistance was estimated by the homeostatic model assessment (HOMA-IR) index: [fasting insulin (mIU/L)]×[fasting plasma glucose (mmol/L)]/22.5.

Venous blood samples were collected from each participant after an overnight fast of at least 8h between 0730 and 1030 h. The blood samples were collected into vacuum tubes with anticoagulant sodium fluoride and centrifuged within 1 hour after collection. Then the blood samples were stored at -20°C immediately and shipped to one central laboratory within 2-4hours, which was certified by the College of American Pathologists. HbA1c was assessed by high-performance liquid chromatography (MQ-2000PT, China). Plasma glucose and lipid profile including triglycerides, high-density lipoprotein (HDL) and low-density lipoprotein (LDL) were measured by BECKMAN COULTER AU 680 (Germany). Fasting insulin was assessed by chemiluminescence (Abbott i2000 SR, USA).TT was assessed by chemiluminescence method (SIEMENS immulite 2000, Germany). FT was measured by enzyme linked immunoassay (BIOTEK ELx808, USA).SHBG was obtained by electrochemiluminescence immunoassay (ROCHE E601, Switzerland).

### Definition of variables

All the subjects were divided into three groups: normal glucose regulation (NGR) group, prediabetes group and diabetes group, in accordance with ADA 2014 criteria. Prediabetes was defined as fasting plasma glucose (FPG) 5.6–6.9mmol/L or HbA1c concentrations between 5.7% (39mmol/mol) and 6.4% (46mmol/mol), or both. Diabetes was diagnosed if the patient had a prior history of diabetes, or if the following criteria was met: FPG≥7.0mmol/L, or HbA1c ≥6.5% (48mmol/mol), or both [14]. As shown in previous research, both economic development status and residence area affected diabetes prevalence in China so that the two variables were taken as covariates in this study [1]. Current economic status was assessed by gross domestic product (GDP) per capita of 2013 in each study site. The mean national GDP per capita (6807 US dollars from World Bank) in 2013 was considered as the cutoff point for economic status.

### Statistical analysis

Continuous variables were presented as median with interquartile range (IQR). To compare the characteristics among the three groups with different glycemic status, Kruskal–Wallis test was used for non-normally distributed continuous data. Analysis of covariance of TT, SHBG and FT (all were log-transformed) in age subgroups was performed among the three groups after adjustment for age because of the association between age and androgens as well as the significant interaction effect of age and different glycemic status.

TT, FT and SHBG were divided into quartiles, with the first quartile representing the lowest one and the fourth quartile the highest. The odds ratios (ORs) and 95% confidence intervals (CI) were calculated using multinomial logistic regression to reveal the risk of diabetes and prediabetes for each quartile of TT, FT and SHBG, with the highest quartile as the reference. Five statistical models were used for multivariate analyses: Model 1: adjusted for age, residence area and economic status; Model 2: adjusted for model 1 and waist circumference. Because BMI and w1aist circumference were highly correlated, we only used waist circumference as the measure of adiposity [19–21]; Model 3: adjusted for model 2 and metabolic factors (LDL, HDL, triglycerides and systolic blood pressure); Model 4: adjusted for model 3 and *a* (FT and TT); or *b* (FT and SHBG); or *c* (SHBG and TT); Model 5 (fully adjusted model): adjusted for model 4 and HOMA-IR.

To further investigate the relationship between serum SHBG, TT and the presence of prediabetes and diabetes in Chinese male populations in age subgroups, the multinomial logistic regression was performed in age strata (<65 and≥65 years), according to the criteria that age≥65 years was defined as elderly men.

All statistical analysis were performed using IBM SPSS Statistics, Version 22 (IBM Corporation, Armonk, NY, USA) and a two-sided P value<0.05 was taken to indicate a significant difference.

## Results

### The characteristics and univariate analyses

A total of 2654 male participants (52.6±13.4 years old) were studied, of whom, 1405 (52.9%) were NGR, 907 (34.2%) were prediabetic and 342 (12.9%) were diabetic. The characteristics of the three groups are listed in Table 1. In Kruskal–Wallis test, male participants with diabetes had significantly lower levels of serum TT compared to NGR and prediabetic groups (*P* <0.001). Compared with NGR and diabetic groups, serum SHBG was detected significantly higher in prediabetic group. The levels of serum FT in NGR group were significantly higher than that of both prediabetic group and diabetic group (*P* <0.001).

**Table 1:** Characteristics of the 2654 male subjects.

	NGR	Prediabetes	Diabetes
Case number	1405	907	342
Age (years)[min,max]	49(21)[18,82]	58(16)[24,93] ^#^	58(13)[30,87] ^#^
TT (nmol/L)	15.6(6.6)	15.1(7.1) *	13.6(6.1) ^#^
SHBG (nmol/L)	38.7(29.1)	42.8(30.7) ^#^^,^ *	36.3(25.8)
FT (pg/ml)	13.5(8.3)	12.2(8.5) ^#^	12.3(9.7) ^##^
HbA1c (%)	5.1(0.5)	5.6(0.7) ^#^^,^ *	6.6(1.7) ^#^
FPG (mmol/L)	5.06(0.54)	5.80(0.60) ^#^^,^ *	7.47(2.36) ^#^
Fasting insulin (mIU/L)	4.11(3.08)	4.49(3.79) ^#^	4.68(4.93) ^#^
HOMA-IR	0.92(0.69)	1.15(1.01) ^#^^,^ *	1.62(1.92) ^#^
BMI (kg/m^2^)	24.1(4.36)	24.7(4.47) ^#^^,^**	25.3(4.73) ^#^
WC(cm)	81.0(12.0)	83.0(13.0) ^#^^,^ *	87.0(13.0) ^#^
LDL (mmol/L)	2.84(0.89)	2.94(0.94) ^##^	2.97(1.03) ^##^
HDL (mmol/L)	1.33(0.38)	1.36(0.43) ^##^^,^**	1.29(0.43)
Triglyceride (mmol/L)	1.33(1.01)	1.50(1.13) ^#^^,^**	1.69(1.49) ^#^
Systolic pressure (mmHg)	127(24)	134(26) ^#^	137(33) ^#^
Residence area [rural/urban (%)]	47.7/52.3	60.4/39.6^#^^,^*	45.9/54.1^&^
Economic status [low/high (%)]	29.9/70.1	23.7/76.3^#^^,^*	22.2/77.8^&^

NGR, normal glucose regulation; TT, total testosterone; SHBG, sex hormone-binding globulin; FT, free testosterone; HbA1c, glycated hemoglobin; FPG, fasting plasma glucose; BMI, body mass index; WC, waist circumference; LDL,low-density lipoprotein; HDL,high-density lipoprotein.

*#* Vs NGR *P*<0.001

*##* Vs NGR *P*<0.05

* Vs Diabetes *P*<0.001

** Vs Diabetes *P*<0.05

& Vs NGR *P*<0.017.

### Analysis of covariance in age subgroups

To control the influence of age, the further analysis of covariance in age subgroups was performed (Table 2). In diabetic group, both TT and SHBG were significantly lower than those of participants with NGR in all three age subgroups. However, in prediabetic group, serum SHBG not only maintained significantly lower than that of NGR group but also was higher than that of diabetic group in all three age subgroups. While, serum TT of prediabetic group was lower than that of NGR group only in <40 years subgroup and significantly higher than that of diabetic group in other two age subgroups. No statistically significant difference for FT was detected in any age subgroup.

**Table 2:** Analysis of covariance of TT, SHBG and FT among three groups with different glycemic status in age subgroups.

	NGR	Prediabetes	Diabetes	P value
TT(nmol/L)				
<40 years	15.7±4.5	13.1±3.5^#^	12.1±5.5 ^##^	<0.05
(case number)	(397)	(71)	(11)	
40–65 years	16.4±6.0	16.0±5.6 *	14.1±5.0 ^#^	<0.001
(case number)	(832)	(583)	(244)	
≥65 years	18.5±6.3	17.5±6.9*	15.2±6.2^#^	<0.01
(case number)	(176)	(253)	(87)	
SHBG(nmol/L)				
<40 years	31.3±15.7	25.6±11.0^#^^,^**	19.3±15.3 ^##^	<0.001
(case number)	(397)	(71)	(11)	
40–65 years	45.1±21.7	43.9±20.3^##^^,^**	38.4±19.3 ^##^	<0.001
(case number)	(832)	(583)	(244)	
≥65 years	71.2±29.9	63.5±29.1^##^^,^**	58.2±30.1^#^	<0.001
(case number)	(176)	(253)	(87)	
FT (pg/ml)				
<40 years	16.4±7.4	15.9±8.7	21.0±7.2	>0.05
(case number)	(397)	(71)	(11)	
40–65 years	14.7±9.5	14.5±8.3	14.2±8.0	>0.05
(case number)	(832)	(583)	(244)	
≥65 years	11.1±5.3	10.5±5.7	11.5±6.7	>0.05
(case number)	(176)	(253)	(87)	

NGR, normal glucose regulation; TT, total testosterone; SHBG, sex hormone-binding globulin; FT, free testosterone.

TT, SHBG and FT were log-transformed due to non-normally distributed continuous data.

adjusted factor: age.

*#* Vs NGR *P*<0.001

## Vs NGR *P*<0.05

* Vs Diabetes *P*<0.001

** Vs Diabetes *P*<0.05.

### Multinomial logistic regression

Among 2654 male participants, increasing quartiles of either SHBG or TT were inversely associated with the presence of both prediabetes and diabetes after adjustment for age, residence area and economic status (*P* for trend<0.0001, Table 3, model 1). After further adjustment for waist circumference (Table 3, model 2) and metabolic factors (LDL, HDL, triglycerides and systolic pressure) (Table 3, model 3), the associations between serum SHBG or TT and the presence of both prediabetes and kinds of androgen values (serum FT and TT for SHBG analysis, FT and SHBG for TT analysis) (Table 3, model 4) and HOMA-IR (Table 3, model 5), the associations between SHBG and the presence of both prediabetes and diabetes were attenuated but remained significant, while the association between TT and the presence of either prediabetes or diabetes was no longer statistically significant.

**Table 3:** Associations of SHBG, FT and TT with prediabetes and diabetes in 2654 male subjects.

Quartiles of sex hormone	Model 1	Model 2	Model 3	Model 4	Model 5
Prediabetes					
**SHBG (nmol/L)**^***a***^					
Q1 (4.7–27.8)	2.39(1.78–3.21)	2.06(1.51–2.82)	1.89(1.38–2.61)	1.82(1.26–2.62)	1.62(1.12–2.36)
Q2 (27.81–40.5)	1.71(1.30–2.24)	1.55(1.18–2.05)	1.47(1.11–1.96)	1.42(1.04–1.94)	1.38(1.00–1.90)
Q3 (40.01–57.03)	1.53(1.19–1.98)	1.45(1.12–1.88)	1.40(1.08–1.82)	1.37(1.04–1.81)	1.41(1.07–1.88)
Q4 (57.031–181.0)	1.0	1.0	1.0	1.0	1.0
*P* for trend	<0.0001	<0.0001	0.00018	0.003	0.026
**TT(nmol/L)**^***b***^				
Q1 (0.35–12.3)	1.73(1.33–2.24)	1.52(1.17–1.99)	1.36(1.04–1.80)	0.97(0.69–1.37)	0.82(0.57–1.16)
Q2 (12.31–15.2)	1.44(1.12–1.85)	1.31(1.02–1.70)	1.25(0.96–1.62)	0.95(0.71–1.29)	0.89(0.65–1.21)
Q3 (15.21–19.0)	1.04(0.81–1.35)	1.00(0.77–1.29)	0.95(0.73–1.23)	0.79(0.59–1.04)	0.77(0.58–1.02)
Q4 (19.01–55.5)	1.0	1.0	1.0	1.0	1.0
*P* for trend	<0.0001	0.0004	0.007	0.716	0.491
**FT(pg/ml)**^***c***^				
Q1 (0.1–9.1)	0.88(0.668–1.14)	0.88(0.68–1.15)	1.00(0.76–1.30)	0.94(0.70–1.27)	0.92(0.68–1.25)
Q2 (9.11–13.0)	0.76(0.59–0.98)	0.77(0.59–0.99)	0.86(0.66–1.12)	0.82(0.62–1.08)	0.84(0.63–1.11)
Q3 (13.01–17.7)	0.75(0.58–0.97)	0.76(0.59–0.98)	0.83(0.64–1.09)	0.81(0.62–1.06)	0.79(0.60–1.04)
Q4 (17.61–146.6)	1.0	1.0	1.0	1.0	1.0
*P* for trend	0.36	0.37	0.949	0.74	0.728
Diabetes					
**SHBG(nmol/L)**^***a***^					
Q1 (4.7–27.8)	5.28(3.50–7.97)	3.63(2.35–5.60)	3.23(2.07–5.04)	2.18(1.30–3.64)	2.09(1.22–3.59)
Q2 (27.81–40.5)	3.08(2.11–4.50)	2.44(1.65–3.60)	2.27(1.53–3.38)	1.68(1.08–2.61)	1.72(1.08–2.73)
Q3 (40.01–57.03)	1.62(1.10–2.37)	1.40(0.94–2.06)	1.37(0.92–2.03)	1.12(0.74–1.70)	1.25(0.81–1.92)
Q4 (57.031–181.0)	1.0	1.0	1.0	1.0	1.0
*P* for trend	<0.0001	<0.0001	<0.0001	0.001	0.004
**TT(nmol/L)**^***b***^					
Q1 (0.35–12.3)	4.61(3.14–6.78)	3.48(2.34–5.18)	3.01(2.00–4.53)	2.15(1.30–3.54)	1.64(0.98–2.76)
Q2 (12.31–15.2)	2.64(1.77–3.93)	2.17(1.45–3.24)	2.03(1.35–3.06)	1.54(0.97–2.44)	1.45(0.90–2.33)
Q3 (15.21–19.0)	1.90(1.27–2.84)	1.71(1.14–2.58)	1.64(1.09–2.47)	1.34(0.87–2.08)	1.32(0.84–2.07)
Q4 (19.01–55.5)	1.0	1.0	1.0	1.0	1.0
*P* for trend	<0.0001	<0.0001	<0.0001	0.002	0.074
**FT(pg/ml)**^***c***^				
Q1 (0.1–9.1)	0.95(0.66–1.34)	0.93(0.65–1.33)	1.11(0.77–1.62)	0.80(0.53–1.20)	0.75(0.49–1.16)
Q2 (9.11–13.0)	0.60(0.42–0.87)	0.60(0.42–0.88)	0.73(0.50–1.08)	0.59(0.40–0.89)	0.62(0.41–0.95)
Q3 (13.01–17.7)	0.82(0.58–1.17)	0.83(0.58–1.19)	0.98(0.68–1.42)	0.88(0.61–1.28)	0.84(057–1.25)
Q4 (17.61–146.6)	1.0	1.0	1.0	1.0	1.0
*P* for trend	0.48	0.437	0.856	0.126	0.113

95% CI are shown in parentheses.

SHBG, sex hormone-binding globulin; TT, total testosterone; FT, free testosterone.

Model 1 adjusted for age, residence area and economic status.

Model 2 adjusted for model 1 and waist circumference.

Model 3 adjusted for model 2 and metabolic factors (LDL, HDL, triglycerides and systolic blood pressure).

Model 4 adjusted for model 3 and *a* (FT and TT); or *b* (FT and SHBG); or *c* (SHBG and TT).

Model 5 adjusted for model 4 and HOMA-IR.

Furthermore, we conducted stratified analyses to reveal the associations between serum SHBG, TT and presence of prediabetes in subgroups of age. Among the male participants ≥65 years old, there was a strong, graded, inverse association between SHBG quartile and the presence of prediabetes after age, residence area, economic status and waist circumference were adjusted (*P* for trend = 0.0001, Table 4, model 2). Based on model 2, further adjustment for metabolic factors (LDL, HDL, triglycerides and systolic blood pressure) changed the *P* value from 0.0001 to 0.001 (Table 4, model 3). Additional adjustment for serum TT and FT did not significantly attenuate this association further (*P* for trend = 0.003, Table 4, model 4). In the full adjusted model, this association partially weakened but remained statistically significant (*P* for trend = 0.019, Table 4, model 5). On the contrary, in the subgroup of participants aged 65 years or older, the inverse association between serum TT and presence of prediabetes was observed in model 2 and model 3 (*P* for trend≤0.01, Table 4), while, after further adjustment for serum SHBG, FT and HOMA-IR, this association was no longer statistically significant (*P* for trend≥0.05, Table 4, model 4, model 5). However, in the subgroup of subjects <65 years old, neither SHBG nor TT had been detected in significant association with the presence of prediabetes in fully adjusted model. The associations maintained statistically significant just after model 3 for SHBG or model 2 for TT was adjusted (*P* for trend≤0.05, Table 4, model 2, model 3). No significant association was found between serum FT and prediabetes or diabetes, whatever any of the five models was adjusted.

**Table 4:** Associations of SHBG and TT with prediabetes in age subgroup.

Quartile of sex hormone	Model 2	Model 3	Model 4	Model 5
**SHBG (nmol/L)**^***a***^				
age<65				
Q1 (4.7–25.6)	1.78(1.25–2.52)	1.64(1.14–2.34)	1.59(1.05–2.42)	1.45(0.95–2.23)
Q2 (25.61–36.4)	1.29(0.93–1.78)	1.24(0.89–1.72)	1.21(0.84–1.75)	1.19(0.82–1.73)
Q3 (36.41–51.1)	1.37(1.00–1.86)	1.33(0.97–1.82)	1.31(0.94–1.82)	1.33(0.95–1.87)
Q4 (51.11–157.0)	1.0	1.0	1.0	1.0
*P* for trend	0.004	0.017	0.058	0.187
age≥65				
Q1 (15.2–42.93)	5.59(1.70–18.34)	5.68(1.69–19.11)	5.33(1.52–18.64)	4.66(1.24–17.54)
Q2 (42.931–60.01)	2.63(1.39–5.00)	2.43(1.25–4.72)	2.27(1.12–4.60)	1.81(0.87–3.76)
Q3 (60.01–82.6)	1.32(0.82–2.14)	1.25(0.76–2.05)	1.20(0.71–2.02)	1.18(0.69–2.03)
Q4 (826.1–181.0)	1.0	1.0	1.0	1.0
*P* for trend	0.0001	0.001	0.003	0.019
**TT (nmol/L)**^***b***^ age<65				
Q1 (1.0–12.2)	1.38(1.02–1.87)	1.23(0.90–1.68)	0.95(0.64–1.41)	0.79(0.53–1.20)
Q2 (12.21–15.0)	1.23(0.92–1.65)	1.17(0.87–1.58)	0.96(0.68–1.36)	0.89(0.62–1.27)
Q3 (15.1–18.5)	0.98(0.73–132)	0.94(0.69–1.27)	0.82(0.59–1.13)	0.80(0.57–1.12)
Q4 (18.51–55.5)	1.0	1.0	1.0	1.0
*P* for trend	0.013	0.091	0.869	0.450
age≥65				
Q1 (0.35–13.0)	2.30(1.24–4.28)	2.19(1.15–4.17)	1.55(0.71–3.38)	1.23(0.55–2.74)
Q2 (13.01–16.5)	1.64(0.94–2.89)	1.57(0.88–2.80)	1.14(0.59–2.21)	1.07(0.54–2.12)
Q3 (16.51–21.4)	0.95(0.57–1.58)	0.87(0.52–1.47)	0.70(0.40–1.24)	0.68(0.38–1.22)
Q4 (21.41–55.5)	1.0	1.0	1.0	1.0
*P* for trend	0.004	0.01	0.216	0.478

95% CI are shown in parentheses.

SHBG, sex hormone-binding globulin; TT, total testosterone.

Model 2 adjusted for age, residence area, economic status and waist circumference.

Model 3 adjusted for model 2 and metabolic factors (LDL, HDL, triglycerides and systolic blood pressure).

Model 4 adjusted for model 3 and *a* (FT and TT); or *b* (FT and SHBG).

Model 5 adjusted for model 4 and HOMA-IR.

## Discussion

In this study, we found that compared to normal controls, Chinese male diabetic patients had significantly lower serum SHBG and TT levels, while Chinese male prediabetic patients had significantly lower serum SHBG. Both low SHBG and low TT levels were associated with increasing presence of prediabetes and diabetes, whereas only the association between serum SHBG and prediabetes and diabetes maintained statistically significant in fully adjusted model, especially in elderly prediabetic men.

The association between androgen and diabetes has been investigated previously. Multiple epidemiological studies showed that low testosterone was found in 30–50% of men with T2DM [6] and might predict the future development of T2DM. The causative relationship between low testosterone and T2DM, which was interrelated with advanced age, obesity, metabolic syndrome and other factors, might be bidirectional or multidirectional [10, 22, 23]. In previous studies, obesity was not only strongly associated with diabetes but also inversely related with the blood testosterone levels in men [24]. A recent study suggested that the intrinsic testosterone could increase the energy consumption and reduce fat accumulation, while, the impairment of testosterone might be related to obesity and metabolic syndrome (MetS) and then increase the risk of T2DM [25]. On the other hand, testosterone supplement could decrease waist circumference, BMI as well as improve serum glucose, HbA1c, lipid profiles and blood pressure in obese hypogonadal men with or without T2DM [26]. It was shown that TT might be the strongest androgen marker that was inversely correlated with many of the metabolic markers related to obesity and insulin resistance [25].

However, in previous studies, the possible roles of various androgen markers were not completely consistent. As shown in a 9-year follow up among elderly men in Finland, it was the higher levels of TT and FT that might independently predicted a reduced risk of T2DM [27], whereas the results of the association between serum FT and the development of T2DM were inconsistent in several longitudinal studies [9, 19, 28]. Both a meta-analysis of 20 cross-sectional studies including 850 men with diabetes and 2,000 normal controls by Ding et al. [29] and another meta-analysis of 28 cross-sectional studies including a total of 1,822 men with diabetes and 10,009 normal controls by Corona et al.[30] showed that it was not FT but TT levels that were significantly lower in diabetic men compared with non-diabetic controls after adjustment for age and BMI. While, in a cross-sectional study of 1,292 middle-aged and older men from England, the association between low testosterone and SHBG concentrations and glycemia was found [12]. Likewise, both low testosterone and low SHBG levels indicated an increased risk of T2DM in men in several earlier studies [8, 9, 31]. However, the roles of TT and SHBG in T2DM remain controversial. Tanabe et al. found that SHBG was significantly inversely associated with waist circumference, BMI, blood insulin levels, HOMA-R and HOMA-β, as well as a significantly lower SHBG level was observed in subjects with MetS compared with those without MetS, suggesting low SHBG level might be a risk factor for MetS [25]. Previous study also showed that it was not serum TT but SHBG was the most powerful predictor of T2DM in men, which predicted T2DM independently of TT [27, 31]. As SHBG-bound testosterone constituted the largest portion of TT, it was inferred that SHBG might be the primary determinant of the apparent relationship between testosterone levels and T2DM [27]. While, on the contrary, the study by Hou et al. showed that the association of TT with glucose metabolism remained significantly after adjusting for SHBG [32].

There were few epidemiologic studies that investigated the relationships between androgens and prediabetes in men, especially in Chinese male adults with the new diagnostic criteria by ADA. Ho et al. indicated that not free or bioavailable T but TT was significantly associated with prediabetes in men [17]. In our study, unlike TT or FT, SHBG of prediabetic patients was found to be not only significantly lower than that of participants in NGR group but also statistically higher than that of diabetic patients in all three age subgroups. In the further multinomial logistic regression analysis, similar to the result of diabetes group, TT and SHBG were inversely associated with the presence of prediabetes. The inverse association between SHBG and presence of prediabetes was weaker but still statistically significant in all of the adjusted models (Table 3), even after further adjustment for TT and FT (Table 3, model 4). While, the association between TT and prediabetes did not persist after adjustment for SHBG and FT (Table 3, model 4). Because of the influence of age on androgen, we further investigated the relationship between SHBG and prediabetes in two age subgroups. The results showed that it was in elderly prediabetic patients that this relationship maintained statistically significant in all the adjusted models, compared to Chinese prediabetic male patients <65 years old. So, in our study, it indicated that, among the three androgen markers, serum SHBG was the most relevant factor associated with diabetes and prediabetes. Low SHBG might be an independent predictor of incident prediabetes in Chinese male adults, especially in elderly men and this relationship might be independent from TT. Besides modulating the biologic effect of testosterone on peripheral tissue, SHBG might participate in glucose metabolism impairment by several potential mechanisms independent of sex hormone. Genetic studies showed that specific SHBG SNPs might influence the risk of T2DM [33]. Carriers of rs6259 polymorphism had higher serum SHBG with a lower risk of T2DM, whereas rs6257 SNP carriers had lower SHBG levels as well as an increased risk of T2DM [34]. Traditionally, BMI was thought to be the independent predictor of circulating SHBG level and hyperinsulinemia, which was caused by insulin resistance and resulted in the impairment of SHBG synthesis in the liver. However, in recent years, in vitro studies showed that under more physiological conditions, insulin did not regulate SHBG production in HepG2 cells [11, 35], as well as in vivo study, using hSHBG transgenic mice, showed that insulin did not downregulate SHBG level either [11]. Recent studies suggested that it was not visceral fat or total body fat but liver fat content that was the major determinant of serum SHBG levels in human subjects [36, 37] and SHBG might be one of the candidates linking nonalcoholic fatty liver disease (NAFLD) which was involved in the pathogenesis of diabetes [38]. Further studies are needed to determine the potential role of low SHBG in the development of prediabetes and diabetes.

The study had some strengths. First, the novelty, it is the first study to investigate the association between serum androgen and the presence of prediabetes and diabetes diagnosed with the new criteria by ADA in Chinese male populations and then to assess which kind of androgen value was the most related marker. Second, the large number of participants of all ages, a total of 2654 Chinese male participants ranged from 18 to 93 years old were recruited into this study. Third, our data source is SPECT-China study that was performed in a general population as opposed to a clinic-based population, so the results may be more reflective.

However, several limitations must be considered. First, owing to the cross-sectional study nature, no causal inference can be drawn. Prospective studies are needed to clarify the precise interrelationship. Second, because of the inconvenience, oral glucose tolerance test (OGTT) could not be performed in these subjects, which might lead to missed diagnosis of diabetes or prediabetes in a part of participants and misclassification of these individuals. Third, a single laboratory measurement of serum testosterone, it has been reported that about 30% of men with low testosterone levels may have normal levels on repeat measurements [39]. Moreover, other confounders, some possible confounders which might bias the relationship between androgen and diabetes or prediabetes, including drugs, systemic diseases, inflammatory and behavioral factors have not been fully assessed and adjusted in this study [21, 40].

In conclusion, Serum androgen was inversely associated with prediabetes and diabetes in Chinese men. Among various androgen values, low serum SHBG was the most relevant factor for prediabetes and diabetes, and whether it is an independent predictor for incident prediabetes in Chinese men needs further explorations.

## Supporting Information

S1 Checklist Strobe checklist for this cross sectional study. (DOCX)
